# Multiple Myeloma Laboratory Diagnostics Made Simple: Practical Insights and Key Recommendations

**DOI:** 10.3390/jcm14197115

**Published:** 2025-10-09

**Authors:** Ana Marta Pires, João Pedro Barreto, Joana Caetano, Maria José Soares, Catarina Geraldes, Bruno Fernandes, Margarida Coucelo, Sérgio Chacim, Henrique Coelho, Cecília Correia, Ana Paula Cruz, Manuel Cunha, Maria Rosário Cunha, Nuno Cunha, Patrícia Ferraz, José Guilherme Freitas, Rui Henrique, Susana Lisboa, Paulo Lúcio, Artur Paiva, Cláudia Pedrosa, Inês Ramos, Ana Bela Sarmento-Ribeiro, Patrícia Seabra, Joana Sevilha, Maria José Rego de Sousa, Sara Sousa, Teresa Sousa, Márcio Tavares, Fernanda Trigo, Adriana Roque, Rui Bergantim, Cristina João, on behalf of the Portuguese Multiple Myeloma Group

**Affiliations:** 1 Clinical Pathology Department, Unidade Local de Saúde Trás-os-Montes e Alto Douro, 5000-508 Vila Real, Portugal; pires.ana.marta@gmail.com (A.M.P.); sara_regueiras_93@hotmail.com (S.S.); 2 Clinical Pathology Department, Instituto Português de Oncologia do Porto Francisco Gentil, 4200-072 Porto, Portugal; jpjpbarreto@gmail.com (J.P.B.); bruno.fernandes@ipoporto.min-saude.pt (B.F.); teresa.sousa@ipoporto.min-saude.pt (T.S.); 3 Hemato-Oncology Unit, Fundação Champalimaud, 1400-038 Lisbon, Portugal; joana.caetano@research.fchampalimaud.org (J.C.); paulo.lucio@fundacaochampalimaud.pt (P.L.); 4 NOVA Medical School, NOVA University of Lisbon, 1169-056 Lisbon, Portugal; mariajsousa@cm-lab.com; 5 Hematology Department, Unidade Local de Saúde São João, 4200-319 Porto, Portugal; mariajoses@gmail.com (M.J.S.); fernandatrigomiranda@hotmail.com (F.T.); 6 Clinical Hematology Department, Unidade Local de Saúde de Coimbra, 3004-561 Coimbra, Portugal; catarinageraldes08@gmail.com (C.G.); absarmento@fmed.uc.pt (A.B.S.-R.); adriroque05@hotmail.com (A.R.); 7 Oncobiology and Hematology Laboratory, Faculdade de Medicina, Universidade de Coimbra, 3004-504 Coimbra, Portugal; 8 Centro Académico Clínico de Coimbra, 3004-504 Coimbra, Portugal; 9 Molecular Hematology Laboratory, Clinical Hematology Service, Unidade Local de Saúde de Coimbra, 3004-561 Coimbra, Portugal; guigascoucelo@gmail.com; 10 Hematology and Bone Marrow Transplantation Service, Instituto Português de Oncologia do Porto Francisco Gentil, 4200-072 Porto, Portugal; schacim@gmail.com (S.C.); inesramos.med@gmail.com (I.R.); 11 Clinical Hematology Department, Unidade Local de Saúde Gaia e Espinho, 4434-502 Vila Nova de Gaia, Portugal; henriquepcoelho@gmail.com (H.C.); maftavares@gmail.com (M.T.); 12 Laboratorial Genetics Department, Instituto Português de Oncologia do Porto Francisco Gentil, 4200-072 Porto, Portugal; ceciliacorreia@ipoporto.min-saude.pt (C.C.); susanalisboa@ipoporto.min-saude.pt (S.L.); 13 Clinical Pathology Department, Unidade Local de Saúde Gaia e Espinho, 4434-502 Vila Nova de Gaia, Portugal; anapaula@chvng.min-saude.pt; 14 Clinical Hematology Department, Unidade Local de Saúde Trás-os-Montes e Alto Douro, 5000-508 Vila Real, Portugal; mmcunha@chtmad.min-saude.pt (M.C.); patricia.morais.ferraz@gmail.com (P.F.); 15 Clinical Pathology Department, Unidade Local de Saúde de Coimbra, 3004-561 Coimbra, Portugal; rosariocunha@chuc.min-saude.pt; 16 Clinical Pathology Department, Instituto Português de Oncologia de Coimbra Francisco Gentil, 3000-075 Coimbra, Portugal; nunocunha@ipocoimbra.min-saude.pt; 17 Hematology Department, Unidade Local de Saúde de Braga, 4710-243 Braga, Portugal; jose.p.freitas@ulsb.min-saude.pt; 18 Pathological Anatomy Department & Cancer Epigenetics and Biology Group, Instituto Português de Oncologia do Porto Francisco Gentil, 4200-072 Porto, Portugal; henrique@ipoporto.min-saude.pt; 19 Porto Comprehensive Cancer Center Raquel Seruca (Porto.CCC) & RISE@CI-IPOP (Health Research Network), 4200-072 Porto, Portugal; 20 Abel Salazar Biomedical Sciences Institute, Universidade do Porto, 4050-313 Porto, Portugal; 21 Operational Management Unit in Cytometry, Unidade Local de Saúde de Coimbra, 3004-561 Coimbra, Portugal; artur.paiva@chuc.min-saude.pt; 22 Coimbra Clinical and Biomedical Investigation Institute (iCBR), Faculdade de Medicina, Universidade do Coimbra, 3004-504 Coimbra, Portugal; 23 Laboratório de Ciências Biomédicas, Escola Superior de Tecnologia da Saúde de Coimbra, Polytechnic Institute of Coimbra, 3045-093 Coimbra, Portugal; 24 Clinical Hematology Department, Unidade Local de Saúde Santo António, 4050-011 Porto, Portugal; claudiamlpedrosa@gmail.com (C.P.); patriciamrseabra@gmail.com (P.S.); 25 Unidade Local de Saúde Médio Ave, 4780-371 Santo Tirso, Portugal; joanabarbosasevilha@gmail.com; 26 Germano de Sousa Clinical Laboratory Center, 1600-513 Lisbon, Portugal; 27 Faculdade de Medicina, Universidade Católica de Portugal, 1649-023 Lisbon, Portugal; 28 Physiology Institute, Faculdade de Medicina, Universidade de Coimbra, 1649-023 Coimbra, Portugal; 29 Cancer Drug Resistance Group, Institute of Molecular Pathology and Immunology (IPATIMUP), Universidade do Porto, 4200-135 Porto, Portugal; 30 i3S—Institute for Research and Innovation in Health, Universidade do Porto, 4200-135 Porto, Portugal

**Keywords:** multiple myeloma, laboratory methodologies, diagnosis, risk stratification, response assessment

## Abstract

Multiple myeloma is a clonal plasma cell malignancy with a highly variable range of clinical manifestations. Over recent decades, substantial progress has been made in laboratory diagnostics, which has deepened our understanding of disease biology, improved risk stratification, and informed treatment strategies. In an era of transformation and innovation, conventional laboratory methods remain essential, as cutting-edge technologies might not be immediately accessible to all laboratories. Nonetheless, even widely used laboratory methodologies present many challenges, such as variability in assay performance, interpretative criteria, and standardization. This review by the Portuguese Multiple Myeloma Group of the Portuguese Society of Hematology provides a comprehensive overview and practical appraisal of current conventional laboratory methods employed for multiple myeloma diagnosis.

## 1. Introduction

Multiple myeloma (MM) is a malignant plasma cell disorder characterized by the clonal proliferation of plasma cells (PC) within the bone marrow (BM), resulting in a complex and heterogeneous clinical presentation [1]. The diagnostic process begins with clinical suspicion. Primary care and emergency staff are frequently the first to encounter patients with subtle or nonspecific symptoms. Recognizing warning signs, such as unexplained anemia, persistent bone pain, hypercalcemia, renal impairment, or recurrent infections, is critical for timely referral and appropriate laboratory evaluation. The disease spectrum includes asymptomatic precursor states, such as monoclonal gammopathy of undetermined significance (MGUS) and smoldering multiple myeloma (SMM), as well as symptomatic MM. This stage is defined by at least 10% of clonal bone marrow PC (or biopsy-proven bony or extramedullary plasmacytoma) and at least one of the myeloma-defining events, summarized by the SLiM-CRAB criteria. These criteria, in addition to the classic features hypercalcemia (C), renal dysfunction (R), anemia (A), and bone lesions (B), consider biomarker-defined events: percentage of abnormal PC in the BM ≥ 60% (S), ratio of involved to uninvolved free light chains in the blood ≥ 100 (Li), Magnetic Resonance Imaging with more than one focal bone lesion (M) [2]. Given this wide clinical variability, accurate diagnosis and risk stratification require the integration of clinical and imaging findings with a diverse range of laboratory data (Table 1).

Diagnostic delay (the interval from symptom onset to definitive diagnosis) remains a major challenge in MM, with median delays of 4–6 months reported [3]. Systematic reviews estimate a median diagnostic interval of ~108 days and a total interval of ~163 days [4]. Delays beyond 3 months are associated with increased risk of complications, including bone disease and renal impairment [5]. These findings highlight the necessity of timely recognition and efficient diagnostic pathways to reduce morbidity and improve outcomes.

In this review, we focus on routinely widely used laboratory methodologies that, when applied to blood, urine, and bone marrow, help in identifying, quantifying, and characterizing monoclonal proteins (MPs) and neoplastic PC.

In recent decades, laboratory diagnostics in MM have undergone changes that have enhanced sensitivity, specificity, and clinical applicability. Traditional core techniques such as serum protein electrophoresis (SPE), serum and urine immunofixation electrophoresis (sIFE/uIFE), and serum free light chain (sFLC) quantification, remain essential to diagnostic algorithms and are indispensable for both initial detection and longitudinal monitoring of monoclonal gammopathies (and more so as cutting-edge technologies might not be immediately accessible to all laboratories) [6]. However, these methods have intrinsic limitations, including analytical variability, interpretive expertise, and awareness of potential confounding factors such as polyclonal background activity or renal dysfunction, which can obscure or mimic pathological signals.

In parallel, other methodologies such as multicolor flow cytometry (FC) and fluorescence in situ hybridization (FISH) added a new dimension to MM diagnostics and have become essential tools for characterizing clonal burden and detecting cytogenetic abnormalities of prognostic significance, including t(4;14), t(14;16), del(17p), and 1q21 gain [7,8]. These genetic alterations are now integrated into risk-adapted treatment strategies, further emphasizing the potential role of these abnormalities in guiding therapeutic decisions [9]. Despite existing challenges, significant progress has been made toward the standardization of FC and FISH protocols across laboratories, reflecting ongoing efforts to enhance diagnostic accuracy and ensure greater consistency in prognostic assessment.

More recently, next-generation flow (NGF) has been incorporated as a sensitive methodology for measurable residual disease (MRD) assessment in MM, with implications in treatment decisions [10]. Next-generation sequencing (NGS) has enabled high-resolution characterization of somatic mutations in key genes, including KRAS, NRAS, BRAF, and TP53, thereby offering important insights into the molecular biology of MM and its clonal evolution [11].

Although traditional laboratory methodologies are extensively validated and integral to clinical practice, they continue to be influenced by interpretative differences and analytical variability. Overall, enhancing the diagnostic landscape in MM will require greater harmonization of protocols, refinement of interpretive criteria, and strengthened multidisciplinary collaboration among clinicians, pathologists, and laboratory scientists. As advancements in automation and cost reduction continue to make both established and emerging techniques more accessible, an increasing number of laboratories are likely to implement them. As such, it remains essential that laboratory staff receive appropriate education to ensure expertise in this field. However, access to these technologies remains uneven across different regions. In underdeveloped countries and underserved areas, limited infrastructure, financial constraints, and a lack of trained personnel pose significant barriers. These disparities can delay diagnosis and compromise patient outcomes. Efforts to promote equitable access, including international collaborations and scalable diagnostic solutions, are essential to ensure that all patients benefit from recent innovations. As MM management continues to evolve toward increasingly personalized approaches, the integration of robust laboratory data into clinical workflows is critical for delivering accurate diagnoses and supporting evidence-based treatment strategies. These changes, along with the emergence of new therapeutic options, have contributed to the development of more advanced laboratory tests.

In this context, the present review, developed by the Portuguese Multiple Myeloma Group of the Portuguese Society of Hematology, aims to provide a comprehensive and practical overview of established laboratory tests and offer a critical appraisal of key methodologies currently employed in MM.

## 2. Immunoglobulins in Multiple Myeloma

Immunoglobulins (Ig) are highly diversified proteins, which reflect their essential role in binding a wide range of foreign antigens in the human body. Each Ig molecule has a characteristic “Y” shape, composed of two identical heavy chains (HC) and two identical light chains (LC). A complete Ig is formed by one of the five HC isotypes—IgA, IgG, IgM, IgD, or IgE—and either a kappa (κ) or lambda (λ) LC. The synthesis of HC and LC is independently regulated by genes located on chromosome 14 for HC, and chromosomes 2 and 22 for κ and λ light chains, respectively [12].

Under normal physiological conditions, the LCs are produced in excess, approximately 10–40% more than the HCs to ensure efficient Ig assembly and prevent the formation of potentially toxic HCs aggregates [13]. The excess LC, known as free light chains (FLC), is released into the bloodstream and primarily cleared by the kidneys. Although there are approximately twice as many PC producing the κ isotype compared to λ, this disproportion is not reflected in the serum FLC ratio (rFLC) in individuals with normal renal function, owing to differences in the renal catabolism rates of the two isotypes [14]. Type κ LC circulates mainly in monomeric form (~25 kDa) and is more efficiently cleared by the kidneys compared to λ LC, which is mainly found in dimeric or oligomeric forms [15].

Understanding the complexity of immunoglobulin synthesis is essential for interpreting variations in the type and concentration of MPs found in pathological conditions and for identifying their function as disease biomarkers.

## 3. Laboratory Methodologies

### 3.1. Serum Protein Electrophoresis

Serum protein electrophoresis (SPE) is a cornerstone methodology in the diagnosis and monitoring of MM, enabling the separation of serum proteins based on their charge-to-mass ratio. Due to its complete automation, increased throughput, and enhanced resolution, particularly between the β1 and β2 fractions, capillary electrophoresis (CE) has largely replaced traditional agarose gel electrophoresis (AGE) in modern laboratories [16]. As discussed in Section 3.2, sIFE complements the information given by SPE and confirms monoclonality [16,17,18].

**Figure 1:**
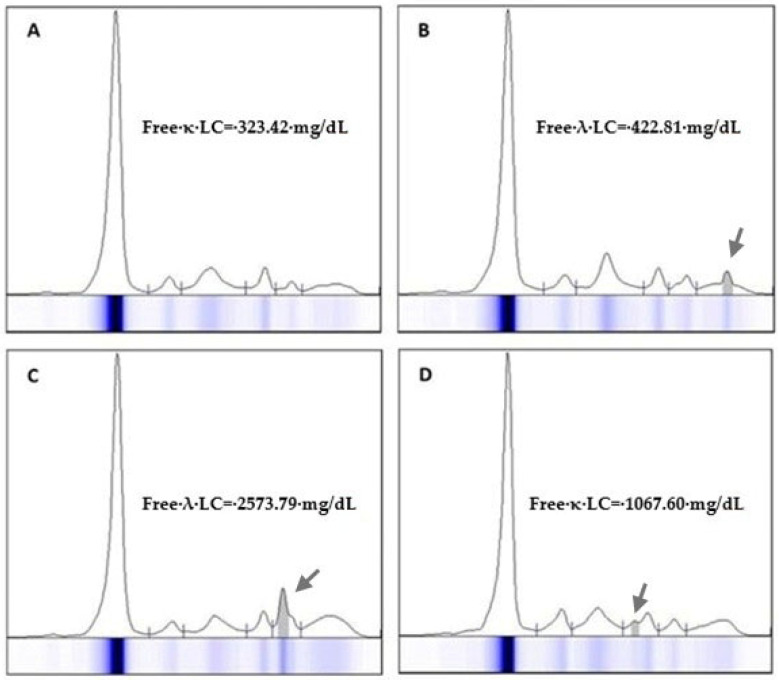
Eletrophoresis of proteins in the serum of 4 patients (**A**–**D**) diagnosed with light chain myeloma. The monoclonal light chain component may migrate in any fraction of the electrophoresis spectrum but is seldom visible at concentrations below 300 mg/dL. Serum free light chain measurement is recommended for the quantification of monoclonal protein in light-chain myeloma. Arrows indicate the migration zone where the free light chain was detected in the electrophoretic trace.

MPs typically migrate in the γ region of the electrophoretic spectrum but can also appear in the β region (especially IgA) [19]. However, any suspicious change in SPE spectrum should be considered. FLC are difficult to detect by SPE but may become visible, especially in cases of high sFLC concentration combined with hypogammaglobulinemia (Figure 1).

Despite methodological improvements, MP quantification remains semi-quantitative, and significant variability can arise from differences in gating strategies and operator interpretation. MP concentration is determined by integrating the monoclonal peak area in the electrophoretic spectrum, a process that is influenced by factors such as the relative peak area, the position of the monoclonal component, and the polyclonal background. Additionally, the accuracy of quantification relies on the operator’s interpretation of the peak’s characteristics and boundaries.

**Figure 2:**
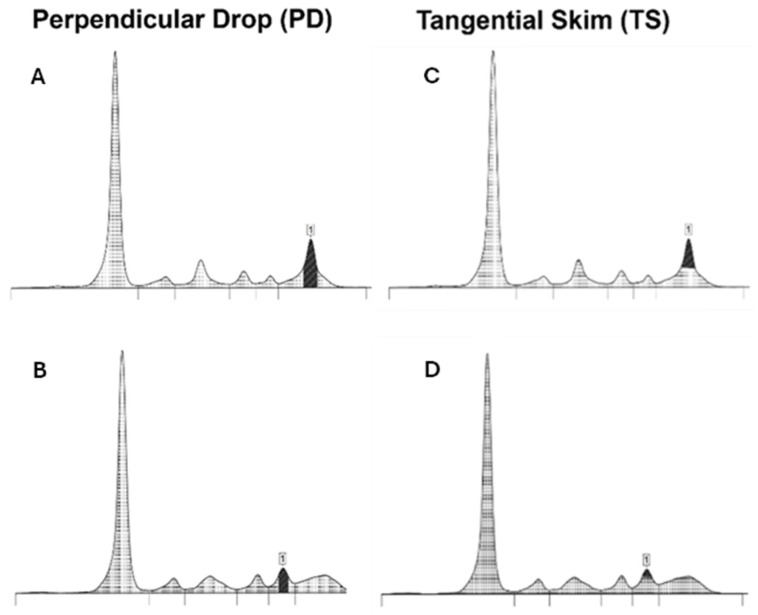
Examples of the perpendicular drop (PD) and tangential skimming (TS) techniques, showing the demarcation of the M-protein peak (**1**). In the PD technique (**A**,**B**), the peak area begins where the M-spike meets the underlying polyclonal region and extends straight down to the baseline. The TS technique (**C**,**D**) includes only the area above the peak by tracing a tangent line along the gamma background. This technique is not reliable if the M-protein migrates in alpha or beta-regions (**D**).

There have been various attempts to develop methods to enhance the precision of M-protein peak measurement in SPE, with perpendicular drop (PD) and tangential skimming (TS) being the most common techniques. PD is the most frequently used technique. In this approach, the demarcation starts where the M-spike meets the underlying polyclonal region and extends straight down to the baseline, encompassing both the area above and below. The TS method attempts to include as M-Protein only the area above the peak. This technique, in contrast to the PD approach, allows for more precise delineation of MP boundaries by tracing a tangent line along the gamma background. TS works well in the γ region but is unreliable for M-proteins migrating in the α- or β-regions, where proteins like transferrin and C3 distort the trace (Figure 2). Both methods have advantages and limitations; however, to ensure reproducibility in serial measurements, it is recommended to apply the same method within the same laboratory [20,21].

The limit of quantification (LOQ) for MPs is not fixed. It varies depending on the location of the MP in the electrophoretic trace (γ vs. β or even α), background immunoglobulin concentration, and electrophoretic system. Katzmann et al. reported that LOQ may range from 1 g/L to >25 g/L (with β-migrating MP being the most challenging to accurately quantify due to co-migrating proteins) [20,22].

Notably, the International Myeloma Working Group (IMWG) defines measurable disease as a serum MP concentration ≥ 10 g/L, which serves as a clinical decision threshold rather than a reflection of analytical sensitivity. MP below this level is technically detectable but may exhibit poor reproducibility, particularly when concentrations fall below 1 g/L. In such cases, quantification should be accompanied by appropriate disclaimers, and results expressed as “<1 g/L” or “trace” where applicable. For this reason, laboratories should adopt method-specific protocols and remain aware of the analytical limitations. Each laboratory is expected to know, validate, and document the limit of detection (LOD) of their SPE system and apply this understanding to result interpretation [21,23].

**Figure 3:**
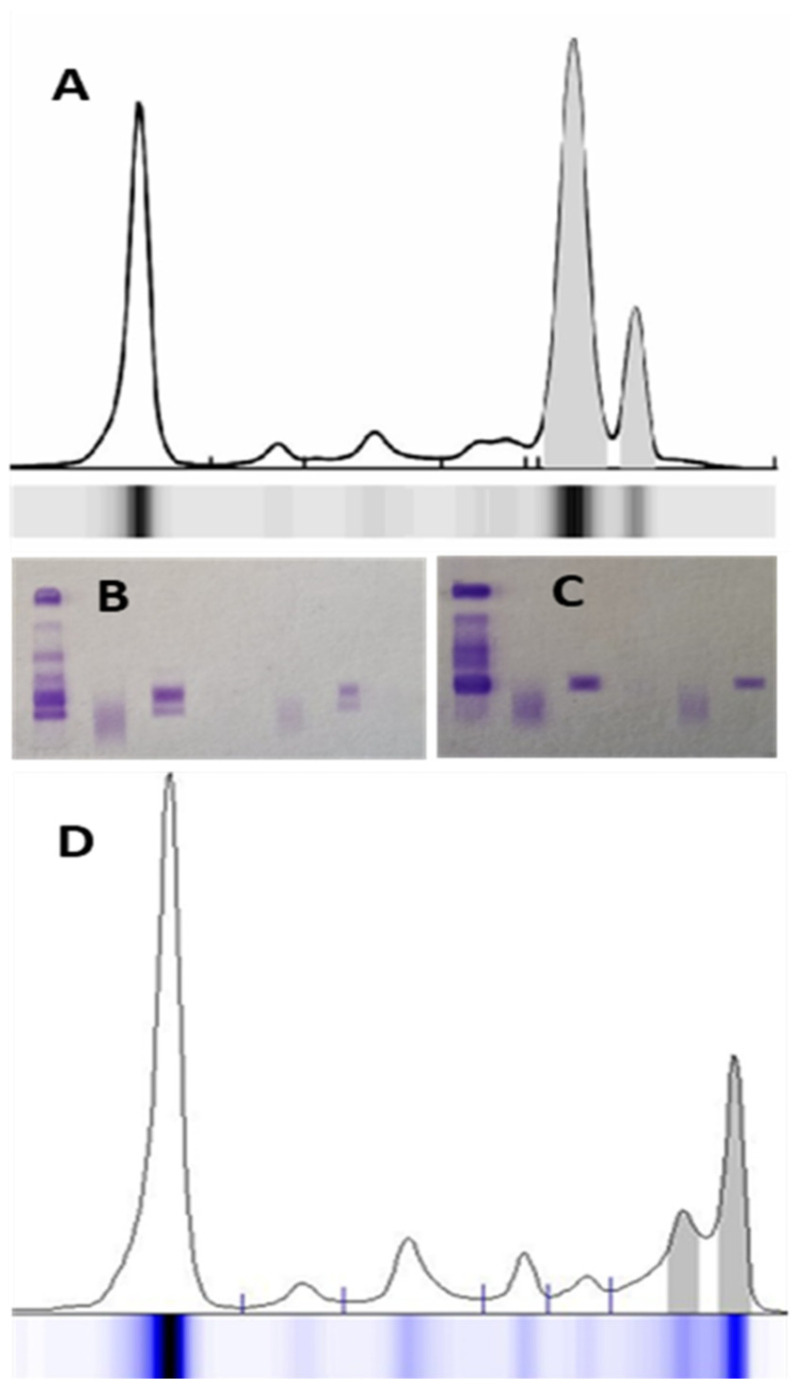
An example of polymerized monoclonal IgAλ protein in serum protein electrophoresis (**A**); the immunofixation (**B**) shows 2 bands corresponding to IgAλ, which, after treatment of the sample with a depolymerizing agent, result in only one band (**C**). Therefore, the total monoclonal protein corresponds to the sum of the areas of the 2 peaks. In another case (**D**), 2 monoclonal components of different Ig isotypes migrate in the gamma zone of the electrophoresis spectrum—IgMλ and IgGλ (from left to right).

Polymerized MP, often associated with IgA or IgM isotypes, can appear as multiple adjacent peaks [20]. In these cases, the peaks must be quantified as a single MP. Only peaks from Ig of different isotypes should be considered independent (Figure 3). The presence of monoclonal Ig is not solely associated with monoclonal gammopathies. The growing application of therapeutic monoclonal antibodies (MoAbs), typically of the IgG subtype (e.g., anti-CD38), may cause subtle alterations in the SPE trace. Other detectable interferences include iodinated radiological contrast agents, antibiotics, hemolysis, and fibrinogen presence [24,25].

**Figure 4:**
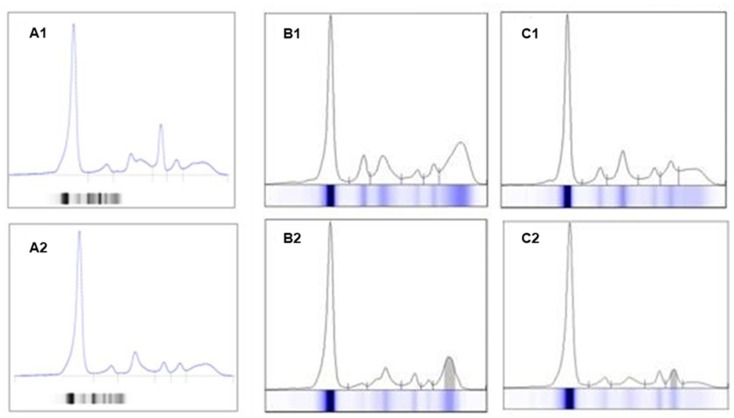
Representation of an electrophoresis spectrum of a sample with hemolysis and fibrinogen interference, with changes in the alpha zone and a peak in beta zone 1 of the spectrum (**A1**) vs. the sample without changes, from a new collection from the same patient (**A2**). Examples of increased polyclonal IgG (**B1**) vs. monoclonal IgGλ protein (**B2**); increased polyclonal IgA (**C1**) vs. monoclonal IgAλ protein (**C2**).

Some other diseases that affect the immune system (e.g., autoimmune diseases, especially rheumatoid arthritis) can cause changes in SPE and sIFE; for this reason, the results must always be evaluated in the patient’s clinical context. The presence of polyclonal Ig in an inflammatory or infectious context is also common; therefore, clonality must be confirmed by sIFE or immunosubtraction (ISUB) (Figure 4).

This variability in electrophoretic patterns, arising from overlapping polyclonal responses, artifacts, or disease-related alterations, underscores the importance of interpretative expertise, particularly when differentiating monoclonal spikes from reactive polyclonal elevations or artifacts. The laboratory professional plays a crucial role in evaluating peak morphology and utilizing confirmatory tests, such as sIFE, to confirm clonality.

In summary, while SPE remains a foundational tool in MM diagnostics, its full clinical utility depends on a detailed understanding of electrophoretic behavior, analytical limitations, and standardization of quantification techniques. Continuous education in interpretation, along with the integration of complementary methods, is essential for maintaining diagnostic accuracy and clinical relevance.

### 3.2. Serum Immunofixation Electrophoresis and Immunosubtraction

Serum immunofixation electrophoresis (sIFE) combines gel electrophoresis with the use of specific antisera that defines the Ig phenotype at diagnosis. sIFE is more sensitive, with a limit of detection (LOD) ≈ 0.1 g/L (0.01 g/dL), when compared to SPE, with LOD ≈ 1.0 g/L (0.1 g/dL). Thus, the confirmation of a complete response requires a negative sIFE [26].

A more recent methodology than sIFE, ISUB combines SPE with the use of specific antisera to identify the Ig isotype. This approach is more automated and efficient, enabling individual sample analysis at a significantly faster pace. Although it has not yet been incorporated into the IMWG recommendations, studies indicate that its sensitivity for detecting quantifiable Ig in SPE is comparable to that of sIFE and it is routinely applied by several laboratories across the world [16,27,28]. However, it is important to note that ISUB demonstrates reduced sensitivity in detecting low concentrations of MPs (those lacking a visible peak in SPE), and FLC, and it lacks specific antisera for identifying IgD and IgE [28]. sIFE remains the reference technique and should be employed whenever ISUB yields inconclusive results, such as negative or ambiguous findings, and/or when meeting IMWG response assessment criteria is required.

The use of sensitive techniques such as sIFE requires considerable operator expertise. Subtle banding patterns may occasionally appear, which are not necessarily indicative of MM. The detection of minor bands, particularly oligoclonal bands following stem cell transplantation, is a frequently observed transient phenomenon reflecting the reconstitution of a restricted clonal immune response [29,30].

Similarly, post-treatment electrophoretic patterns resulting from MoAb therapies, such as daratumumab, elotuzumab, or isatuximab, must be precisely recognized by the operator [31,32]. The primary concern with these therapies lies in their potential to confound response assessment, as therapeutic bands may mimic residual disease and lead to misclassification, particularly mistaking complete response (CR) for very good partial response (VGPR) [33]. To address interference from therapeutic MoAbs, manufacturers are developing targeted solutions to differentiate them from endogenous MPs. One such approach, the Hydrashift 2/4 Daratumumab assay (Sebia), builds upon the principles of the daratumumab-specific immunofixation electrophoresis reflex assay (DIRA); it uses an anti-daratumumab antibody to modify the drug’s electrophoretic migration, enabling a clear distinction from MP [34]. Similar principles are applied to the recent Hydrashift 2/4 Isatuximab assay (Sebia, Lisses, France) [35].

It is important to reinforce that, although ISUB offers an automated and efficient workflow, sIFE remains the gold standard for evaluating equivocal cases or fulfilling IMWG response criteria. For detecting monoclonal proteins accuracy remains operator-dependent and requires specific interpretive expertise. When there is high clinical suspicion of MM without the presence of a monoclonal band in sIFE, we suggest performing the test in a new serum sample. The timing for collecting this new sample should be guided by the patient’s clinical evolution; alternatively, we suggest an interval of 3 months between samples.

### 3.3. Urine Immunofixation Electrophoresis and Urine Protein Electrophoresis

For many years, MP testing in 24 h urine was used to characterize and monitor light chain MM and AL amyloidosis. Several studies suggest that 24 h urine testing can be effectively replaced by sFLC measurement in most light chain MM patients [36,37,38]. sFLC eliminates the need for urine collection, offers greater sensitivity and precision than urine protein electrophoresis (UPE) for MP quantification, and serves as an established prognostic marker [39]. Exceptions include patients with suspected AL amyloidosis or light chain deposition disease, in whom urine immunofixation electophoresis (uIFE) is mandatory [40].

Despite these advances, the IMWG recommendations (dated from 2014) support the use of 24 h urine analysis for diagnosis [2], response assessment [26] and monitoring of MM patients with renal impairment [41]. Quantification of MP in urine should be based on the area under the monoclonal peak on UPE. Although some laboratories measure total urinary light chains to estimate MP levels, no formal recommendations in the literature currently support this approach.

The use of sFLC assay allows for a more selective use of 24 h urine testing; however, in cases of result discrepancies or diagnostic uncertainty, uIFE on a 24 h specimen remains recommended.

### 3.4. Serum Free Light Chains Measurements

MP may be secreted as a complete Ig, complete Ig associated with κ or λ LC, or as FLC alone, which occurs in approximately 20% of cases. The LC neo-epitope is exposed only in the unbound (free) form, which enabled the development of the sFLC assay, for more precise quantification in circulation. Free κ and λ LCs and the free κ/λ ratio (rFLC) must be evaluated simultaneously, as it is the alteration of this ratio that indicates monoclonality [14]. Interpreting these three parameters together enhances diagnostic accuracy. For example, an abnormal rFLC combined with immunoparesis of one LC type is more suggestive of monoclonality than an abnormal ratio accompanied by elevations in both chains. Clinical context and understanding of FLC physiology are essential, as various factors, such as renal dysfunction, inflammatory or reactive conditions, liver disease, and autoimmune disorders, may influence FLC concentrations.

The sFLC assay has significantly advanced the detection and monitoring of FLC-related disease in MM [2], and the rFLC has emerged as a valuable biomarker for both diagnosis and prognosis [42,43].

In pathological conditions, FLC production may vary significantly, both in absolute value and relative to intact Ig; in fact, FLCs and complete Ig are regarded as independent biomarkers [44]. Given the potential for significantly elevated FLC concentrations, laboratories must be prepared to detect antigen excess, which can be readily identified by analyzers equipped for kinetic monitoring [45]. In cases of unexpectedly low results, repeating quantification with higher dilutions is recommended, following the manufacturer’s guidelines.

LOD in sFLC assay can reach approximately 0.1 mg/dL [14,46], making it substantially more sensitive than sIFE (sIFE; LOD ≈ 10 mg/dL) (Appendix A Table A1), particularly in scenarios involving coexisting FLC isoforms or polymerization. Subtle variations or progressive increases in the involved FLC, even when minimal and observed across two to three sequential assessments, may indicate disease progression, changes that are unlikely to be detected by electrophoresis (LOD ≈ 100 mg/dL). Conversely, a decrease in FLC levels provides a more rapid indication of treatment response compared to conventional markers [47].

Patients with renal impairment require special consideration, as rFLC may be affected by abnormal renal clearance. Some studies recommend using sFLC reference ranges adjusted for renal function [48,49].

Since 2014, an rFLC ≥ 100 (with involved FLC ≥ 100 mg/L) has been included in the diagnostic criteria for MM under the SLiM-CRAB framework [2]. rFLC is also used to assess the risk of progression in patients with MGUS according to the by Mayo score (abnormal rFLC < 0.26 or >1.65; rFLC < 8 in LC-MGUS) [44,50] and SMM by 2/20/20 risk stratification model (≥20) [51]. Additionally, rFLC monitoring is valuable in patients with intact immunoglobulin MM, given the potential for relapses through light chain escape [43].

The difference between the involved and uninvolved FLC (dFLC) serves as a biomarker for risk stratification in AL amyloidosis [52,53] and for treatment response assessment in MM [26]. As dFLC is unaffected by suppression of the uninvolved chain, which may distort the rFLC, it offers improved reproducibility in certain clinical settings as AL amyloidosis [53].

Currently, various methodologies are available for sFLC measurement, using either polyclonal or monoclonal antibodies. Comparative studies have demonstrated that these assays are not interchangeable and underscore the need for standardization through the development of an international reference standard [54,55,56].

Method selection remains at the discretion of individual laboratories. However, the clinical decision thresholds cited in this document reflect those endorsed by the IMWG, established and validated using the Freelite^®^ assay (The Binding Site Group Ltd., Birmingham, UK) [57]. Alternative assays, such as N-Latex FLC (Siemens Healthineers, Erlangen, Bavaria, Germany) or Sebia FLC, may be used in clinical practice but differences in calibration, reference ranges, and sensitivity must always be considered when interpreting results. In the event of a methodology switch, given the lack of assay equivalence, a baseline must be established by concurrently testing patient samples with both assays over a defined period to ensure the clinical applicability of the selected method.

### 3.5. Immunoglobulin Measurements

Total Ig quantification typically performed using automated methods such as nephelometry or turbidimetry, measures both monoclonal and polyclonal components and is therefore not recommended for identifying or quantifying MP [19]. Nonetheless, Ig measurement is advised at diagnosis to assess immunoparesis and, in IgA and IgD MM subtypes, can complement SPE, particularly when accurate delineation of the monoclonal peak is challenging. In these scenarios, the same principles applied to MP quantification in SPE should guide Ig monitoring during treatment response assessment [26]. These limitations can be addressed through the measurement of immunoglobulin heavy/light chain (HLC) ratios, enabled by the development of the Hevylite^®^ assay (The Binding Site Group Ltd., Birmingham, UK), which quantifies specific IgGκ/IgGλ, IgAκ/IgAλ, or IgMκ/IgMλ pairs [58,59,60]. Beyond analytical utility, HLC measurement also carries prognostic value by enabling quantification of the uninvolved Ig, thus facilitating immunoparesis evaluation [61,62]. This assay is not yet included in the routine biomarker panel for MM diagnosis. Nevertheless, it has been employed in various studies and clinical trials demonstrating its utility in patient follow-up [63,64], particularly in cases involving MPs that migrate within the β fraction on SPE or appear as multiple peaks due to polymerization [60].

The IMWG has also acknowledged its potential as an alternative method for MP quantification when peak migration occurs in the β zone [26]. Given the methodological differences between SPE and HLC assay, their results are not interchangeable.

### 3.6. Morphological Examination

Peripheral blood morphologic examination has been historically of help in diagnosing MM by identifying characteristic features such as erythrocyte rouleaux formation (due to paraproteins in high concentrations) and the observation of morphologically abnormal PC in circulation (which indicates plasma cell leukemia if above 5% of the leucocyte differential count) [65,66].

BM morphologic examination can be performed in samples obtained by aspiration or core-needle biopsy to identify and quantify infiltration by PC. The samples are usually collected in the posterior superior iliac crest, the breastbone, or, more seldom, the anterior superior iliac crest. At diagnosis, it is recommended that both BM aspiration and biopsy are performed [6], and, in case of differences in counting PC, the highest value should be considered [2].

The BM is usually heterogeneously affected by MM (in patches, or interstitial or diffuse patterns). In 5% of BM aspirations, diagnosis is not possible due to a lack of representativeness or hemodilution. In 5 to 10% of BM biopsies, diagnosis is not possible due to a lack of representativeness or because the disease is in a very early phase [2,67,68]. In these cases, as well as in those with PC below 10% but with a high suspicion of MM, a new aspiration/biopsy sample should be obtained in a different anatomical location or, if there are suggestive lesions, an image-guided biopsy should be performed [2,69].

Plasma cell clonality should be determined by FC in a BM aspiration or by immunohistochemistry in a BM biopsy [68].

In the minority of patients with non-secretory MM (with negative MP by SPE/sIFE, UPE/uIFE, and sFLC), the medullary plasmacytosis, observed by morphologic examination or immunophenotyping by FC, is the only measurable marker both at diagnosis and follow-up [70,71].

An accurate and efficient BM morphological examination depends on the quality of the sample, the smear/section, and coloration (of the Romanowsky type, as May-Grünwald-Giemsa or Wright-Giemsa). An experienced observer should look at a minimum of 200 cells [72,73]. Morphologic features in BM examination are variable and are summarized in Table 2.

**Figure 5:**
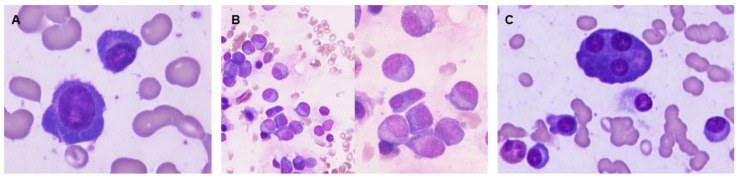
Examples of bone marrow samples colored by May-Grünwald-Giemsa, 1000× magnification. Plasma cell with morphologic immaturity: high nucleus/cytoplasm ratio and dispersed chromatin (**A**). Binucleated plasma cell and several plasma cells (PC) with dispersed chromatin (**B**). Bone marrow showing mature PC and a multinucleated plasma cell (**C**).

Although nuclear morphologic abnormalities, such as binucleated or multinucleated PC, immature PC, or plasmablasts, are not included in diagnostic or staging criteria, their presence has been linked to poorer prognosis and an increased risk of relapse (Figure 5) [68,74].

### 3.7. Flow Cytometry

In 2014, the IMWG specified that while ≥10% clonal PC in the bone marrow defines multiple myeloma, the presence of ≥60% PC was introduced as a myeloma-defining event, indicating symptomatic disease [2]. Flow cytometry (FC) represents a rapid, sensitive, and reliable method to identify and quantify aberrant PC, even when present at small percentages in a BM sample. Applying FC allows for unequivocal distinction of neoplastic PC from their normal counterparts and assessment of plasma cell clonality [76]. It provides important information for diagnosis and classification, while simultaneously contributing to prognostic stratification of MM patients. Previous studies have demonstrated that less than 5% of normal PC among total PC indicate poor outcomes in MM and higher progression risk of MGUS and SMM into symptomatic disease [77], and that specific immunophenotypic profiles are associated with MM outcome [78].

**Figure 6:**
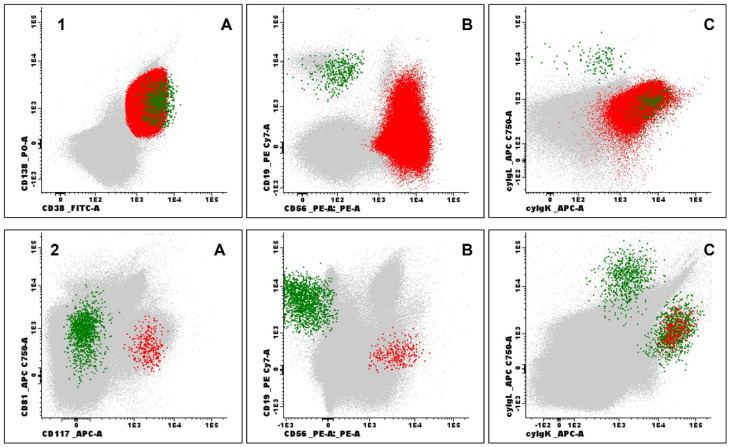
Examples of immunophenotypic evaluation by flow cytometry in patients with multiple myeloma. (**1**) Bone marrow aspirate evaluation at diagnosis: Normal plasma cells (PC) (green) and neoplastic PC (red) based on CD138 (positive) and strong CD38 (positive) expression (**1A**), CD19 (negative) and CD56 (positive) expression (**1B**), cyIgλ (negative) and cyIgκ (positive) expression (**1C**), all other events represented in grey; (**2**) Measurable residual disease by Next Generation Flow: Normal PC (green) and aberrant PC (red) based on CD81 (negative) and CD117 (positive) expression (**2A**), CD19 (negative) and CD56 (positive) expression (**2B**), cyIgλ (negative) and cyIgκ (positive) expression (**2C**). Processing, antibody panels, and acquisition followed the protocols established by the EuroFlow consortium (samples acquired using the FACSLyric^®^ flow cytometer and analyzed with Infinicyt^®^ software version 2.1, both by BDBiosciences, Franklin Lakes, NJ, USA).

The European Myeloma Network (EMN) issued consensus recommendations in 2008 regarding the clinical utility of FC in MM and other clonal plasma cell-related disorders and how to apply it in a routine diagnostic laboratory [7]. In the later 2018 guidelines, and the recent 2025 European Hematology Association (EHA)-EMN evidence-based guidelines, FC was confirmed as a recommended tool for diagnosis in MM [6,79]. Plasma cell clonality is confirmed by intracellular κ (cyIgκ) or λ (cyIgλ) light chain restriction. Aberrant antigen expression patterns are identified using a specific antibody panel that includes markers to precisely identify PC, CD38 and CD138, as well as discriminatory markers between normal and neoplastic cells, including CD19, CD45, CD56, CD117, CD27, CD28, and CD81. The characteristic light scatter of PC should also be considered (Forward Scatter-FSC—representing size and Side Scatter–SSC—representing internal cell complexity or granularity). Neoplastic PC typically exhibit CD38 intermediate/high (dim positive in 80% of MM cases) and CD138-positive expression, with intermediate to high FSC and SSC characteristics, usually negative CD19, CD27 and CD81 (negative or dim positive in 40–50% and 55%, respectively), CD45 (negative in 73%), CD56 (strong positive in 60–75%), CD28 and CD117 (strong positive in 15–45% and positive in 30%, respectively), and beta2-microglobulin cell surface expression (serving as a prognostic marker) (Figure 6) [7,80]. DNA ploidy and cell cycle analysis employing propidium iodide are used to determine ploidy status, as a percentage of S-phase PC above 2% of PC has long been shown to have prognostic value, with shorter disease-free survival and overall survival [81].

Immunophenotypic evaluation by FC should be performed on BM samples of 1–2 mL in dipotassium (K2) or tripotassium (K3) ethylene diaminetetraacetic acid (EDTA)—particularly relevant for staining of the CD138 antigen, whose expression levels on PC are higher in EDTA- vs. heparin anticoagulated samples. These should be processed within 12–24 h post-collection [82],), with measurements of at least 0.5 to 1 × 10^5^ events after excluding platelets and debris/doublets [7]. Discrepancies in plasma cell quantification between BM morphological evaluation and FC usually occur in hypocellular samples, mostly due to the order in which samples are collected and low-quality samples, including coagulated, aged (beyond 48 h), and hemodiluted. Samples lacking hematopoietic precursor populations, such as erythroblasts and myeloid or B-cell precursors, and mast cells should be interpreted with caution, as these populations serve as internal quality controls to exclude hemodilution [83].

Following the advances in therapeutic efficacy, MRD was included in response criteria [26]. Achieving undetectable MRD in patients with CR is one of the most important prognostic factors, as it is associated with longer progression-free survival (PFS) and overall survival (OS) [79,84]. Currently, clinical trials are exploring how therapy adjustments based on MRD status impact patient survival [85]. Sensitivity in MRD assessment by FC has increased with the introduction of the NGF approach developed by the EuroFlow consortium and included in the IMWG criteria [86]. For MRD assessment, the first BM aspirate (first pull) should be used. Bulk lysis processing of at least 10^7^ cells and a standardized two-tube, 8-color antibody panel (including a multi-epitope anti-CD38 antibody to avoid interference in patients treated with anti-CD38 therapy, along with CD138, CD45, CD19, CD56, CD27, CD81 and CD117, cyIgκ and cyIgλ) allows sensitivity levels between 10^−5^ and 10^−6^ to be achieved. The LOD and LOQ depend on the number of total cells evaluated and the number of pathological PC identified (e.g., total number of cells evaluated = 2,000,000; clonal PC = 1000/0.05% of total cells; LOD = 0.0015% and LOQ = 0.0025%) [87]. Populations whose low or absent values indicate hemodilution (such as mast cells, erythroblasts, and myeloid and B lymphoid precursors) should be identified and compared to reference values (Figure 6) [88].

The NGF approach works without the need for a diagnostic sample (i.e., a basal FC characterization), as it identifies clonal PC by detecting immunophenotypic aberrancies. This method efficiently evaluates sample quality while remaining cost-effective. Low sample quality and quantity (coagulated, processed after more than 48 h, or hemodiluted) may result in false negative results. Ongoing innovations, such as automated analysis with a reference database, can enhance the detection of malignant plasma cell populations by reducing analysis time, diminishing operator variability, and facilitating inter-laboratory harmonization, as demonstrated in other hematological malignancies [89,90]. Nevertheless, these automated analysis techniques must be robust and deliver high-quality data output so that results can be reported with confidence.

Despite its strengths, NGF has limitations. As previously stated, sample quality can significantly impact assay performance. Additionally, it remains invasive, requiring BM sampling, which may not always reflect the full spatial heterogeneity of disease, particularly in cases with patchy medullary or extramedullary involvement. Peripheral blood-based detection strategies are being explored. Circulating tumor plasma cells (CTPC) released from primary or metastatic sites into the bloodstream may be responsible for MM spreading and can be used as a biomarker for diagnosis and disease progression, eventually outperforming tumor burden BM quantification in the differentiation between precursor stages of disease and active disease [91,92]. Evaluation of CTPC has prognostic value in MM [93], and several studies have shown its association with stage of disease, disease progression, and even higher relapse rate [94]. Differences in the immunophenotypic pattern of antigen expression between normal PC circulating in peripheral blood and those present in the BM have already been identified in earlier studies, including dimmer expression of CD27, CD38, and CD138 in CTPC, thus impacting their identification [76]. Detection methods for CTPC assessment, like the NGF assay developed and validated by the EuroFlow Consortium, have been used in clinical trial settings but need further testing and standardization [91]. While these approaches are not yet validated for routine clinical use, they promise to make MM diagnosis and MRD assessment by NGF more accessible and less invasive.

### 3.8. Cytogenetics

MM is a genetically heterogeneous disease. Primary cytogenetic abnormalities (CA), which occur early in disease development and are often already present in precursor conditions, are considered the foundational or initiating genetic events of MM [95,96]. Their two main categories are hyperdiploidy and Immunoglobulin Heavy Chain (IgH) translocations, which do not co-occur. Hyperdiploidy is characterized by the presence of extra odd-numbered chromosomes, which are believed to result from mitotic errors that lead to chromosomal gains. This subtype often has a better prognosis than some IgH-translocated subtypes. IgH translocations involve translocations between the IGH locus on chromosome 14q32 and various partner oncogenes, leading to deregulation of gene expression [97]. These include:

- t(4;14)(p16;q32), deregulating the expression of FGFR3 and NSD2;
- t(14;16)(q32;q23), deregulating the expression of MAF;
- t(11;14)(q13;q32), deregulating the expression of CCND1;
- t(6;14)(p21;q32), deregulating the expression of CCND3;
- t(14;20)(q32;q11), deregulating the expression of MAFB.

**Figure 7:**
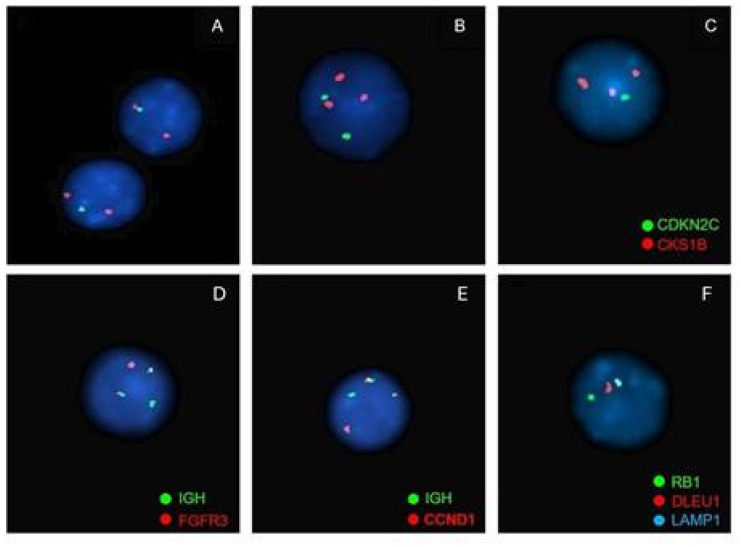
Examples of the most frequent alterations detected by FISH in patients with multiple myeloma. (**1**) Chromosome 1 abnormalities: loss of 1p31 and gain of 1q21. (probe used: XL 1p32/1q21 Amplification/Deletion Probe, MetaSystems—1p32 (gene CDKN2C Spectrum Green) and 1q21 (gene CKS1B Spectrum Orange)) (**A**). Loss of 1p32: 1 green signal and 2 orange signals; (**B**). Gain of 1q21: 2 green signals and 3 or more orange signals; (**C**). Loss of 1p32 and gain of 1q21: 1 green signal and 3 or more orange signals. (**D**) IGH::FGFR3 t(4;14) rearrangement. 2 fusion signals (derived from the translocation), 1 green signal (locus IGH) and 1 orange signal (gene FGFR3) (probe used: Vysis LSI IGH/FGFR3 DCDF Probes, Abbott—locus IGH Spectrum Green and gene FGFR3 Spectrum Orange). (**E**) IGH::CCND1 t(4;11) rearrangement. 2 fusion signals (derived from the translocation), 1 green signal (locus IGH) and 1 orange signal (gene CCND1) (probe used: Vysis LSI IGH/CCND1-XT DCDF Probes, Abbott—locus IGH Spectrum Green and gene CCND1 Spectrum Orange). (**F**) Chromosome 13 abnormalities: Monosomy 13. 1 green signal, 1 orange signal and 1 aqua signal (probe used: XL RB1/DLEU/LAMP Deletion Probe, MetaSystems—gene RB1 Spectrum Green, gene DLEU1 Spectrum Orange and gene LAMP1 Spectrum Aqua).

Their detection provides critical stratification of risk, allowing for more informed therapeutic strategies. For instance, the identification of t(11;14) has gained clinical relevance with the advent of BCL-2 inhibitors, highlighting the utility of cytogenetics for targeted therapy selection, besides prognosis [98]. Secondary cytogenetic events, such as del(17p) (TP53), chromosome 13 monosomy/deletion, and 1q21 gain/amplification, are frequently associated with disease progression and therapeutic resistance, underscoring their role in indicating poor outcomes (Figure 7) [9].

Conventional karyotyping has limited sensitivity in MM because PC have a low proliferative index, resulting in few metaphases. This limitation restricts its clinical utility, relegating it largely to the identification of rare chromosomal abnormalities [99]. Therefore, cytogenetic analysis using FISH on purified CD138-positive PC is the standard method and should be routinely performed in newly diagnosed and overt relapsed cases. Its greater sensitivity allows for better classification and prognostic stratification of MM patients [8]. To ensure consistency and accuracy in interphase FISH, the EMN established a set of technical consensus guidelines intended to harmonize FISH testing across centers, reduce inter-laboratory variability, and support reliable risk stratification in multiple myeloma [8]. These include using preferably the first BM aspiration to optimize sample quality, collected in sodium or lithium heparin, and performing timely processing (within 24 h of collection) to preserve cellular integrity. As PC often constitute a small fraction of BM cells, without enrichment, FISH results may be diluted or misleading. Plasma cell enrichment techniques include Magnetic-Activated Cell Sorting (MACS) using antibodies conjugated to magnetic beads that target CD138, highly expressed on PC, and Fluorescence-Activated Cell Sorting (FACS) using fluorescently labeled antibodies (e.g., CD138, CD38, CD45) to sort PC by FC individually [100]. In the study of plasmacytomas, formalin-fixed, paraffin-embedded tissue is used. The recommended positivity thresholds are ≥10% for translocations detected by fusion or break-apart probes and ≥20% for numerical abnormalities. A standardized probe panel should be employed, using commercially validated reagents, and a minimum of 100 PC should be scored per sample. Each laboratory must establish confidence limits and validate methods using both normal and abnormal samples to define patterns of aberrant signals. A trained analyst should be designated for result interpretation, and reports should clearly state the percentage of abnormal cells, the methodology used, and the type of abnormality detected.

CA that are associated with poorer outcomes, including shorter PFS, OS, and generally worse responses to therapy, represent adverse prognostic markers. Patients with favorable markers are classified as standard or low risk and typically have better long-term outcomes [101]. The major molecular/genomic prognostic markers have been integrated into risk stratification systems such as the Second Revision of the International Staging System (R2-ISS) [102], mSMART [103], and IMWG guidelines [104]. A consensus genomic staging recommendation on the definition of high-risk MM was recently proposed by the International Myeloma Society (IMS)-IMWG, which relies upon the presence of at least one of the following abnormalities that confer higher risk: del(17p), with a cutoff of >20% clonal fraction, and/or TP53 mutation, an IgH translocation including t(4;14), t(14;16), or t(14;20) along with 1q+ and/or del(1p32), or monoallelic del(1p32) along with 1q+ or biallelic del(1p32) (Table 3) [105].

While FISH can reliably identify CA that was previously undetected by conventional cytogenetics, its targeted nature limits detection to known rearrangements, potentially overlooking novel or complex structural variants such as small deletions or cryptic translocations [106]. Also, FISH’s sensitivity is limited by the proportion of PC analyzed and cannot comprehensively capture subclonal heterogeneity, which is increasingly recognized as a driver of treatment failure [107].

### 3.9. Molecular Genetics

Gene expression profiling (GEP) and single-nucleotide polymorphism (SNP) assay-based platforms can accurately detect cytogenetic subtypes and are becoming useful for classification and clinical decision-making [108,109]. More recently, NGS has revolutionized molecular profiling in MM by enabling high-resolution interrogation of the mutational landscape and clonal architecture [110]. NGS can detect a broad spectrum of somatic mutations that appear later, contributing to malignant progression, commonly involving RAS/RAF pathway genes (KRAS, NRAS, BRAF), TP53 mutations indicative of high-risk disease, and alterations in NF-κB signaling components (TRAF3, CYLD), that bear significant prognostic and therapeutic implications [111]. There also appears to be a link between specific mutations and primary CA, such as the KRAS mutation, which is more common in patients with t(11;14), and the co-occurrence of del17p with TP53 mutations, which is associated with a poorer prognosis in relapsed patients [95]. The ability to track clonal evolution longitudinally via NGS provides a dynamic view of disease biology that cytogenetics alone cannot achieve. These capabilities could enhance diagnostic clarity and therapy guidance. Nonetheless, bioinformatic complexities and variability in assay standardization raise concerns regarding reproducibility and inter-laboratory comparability [112]. In the context of diagnosis, NGS is not yet used in routine clinical diagnosis and remains limited to clinical trials.

NGS, along with NGF, has been recognized as a reference method for MRD assessment by the IMWG and the EMN. This involves studying the specific rearrangement pattern of the IgH gene (VDJ, DJ, IgK, and IgL) present in each patient [113]. For NGS-based MRD assessment, the first BM aspiration should be used to optimize sample quality, collected in EDTA, and processed promptly (within 24 h of collection). Generally, 2–3 million PC (CD138-positive) magnetically separated by MACS or sorted by FACS (equivalent to approximately 21.6–27 μg of DNA) are required to achieve a minimum sensitivity of 10^−6^ with 95% confidence in available assays such as clonoSEQ (Adaptive Biotechnologies, Seattle, WA, USA) and LymphoTrack [114]. Although it can be performed on cryopreserved samples, this methodology is demanding in routine practice because relatively large volumes of BM are required for clinical testing. NGS faces other practical challenges, including the requirement of diagnostic baseline samples to establish clonotypes, higher costs, and longer turnaround times compared to NGF [115].

## 4. Discussion

MM represents a biologically and clinically heterogeneous disease that requires a multifaceted diagnostic approach. While the clinical landscape is rapidly evolving with the advent of precision medicine and novel therapies, the integration of traditional and modern laboratory techniques remains essential to achieving diagnostic accuracy and optimizing patient outcomes.

**Figure 8:**
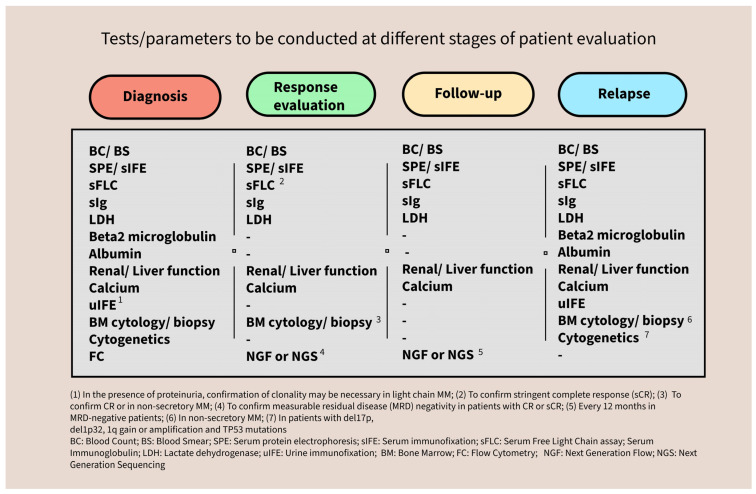
Summary of the tests to be conducted at the different stages of patient evaluation.

Conventional assays such as SPE, sIFE, and sFLC quantification continue to serve as foundational tools in clinical laboratories and are essential for the detection and longitudinal monitoring of monoclonal proteins. Likewise, the clinical value of urine studies, though now more selectively employed, remains relevant in specific contexts. Advanced techniques, including FC and FISH, have significantly enhanced our ability to characterize the immunophenotypic and genetic profiles of neoplastic PC, provide critical prognostic information, and help refine risk-adapted treatment strategies (Figure 8).

Importantly, this review emphasizes that diagnostic precision depends not only on technical sophistication but also on pre-analytical quality, methodological standardization, and interdisciplinary collaboration. The heterogeneity in sample types, collection protocols, and interpretation frameworks underscores the need for harmonization across institutions and national guidelines. Laboratory professionals play a critical role in navigating analytical limitations, identifying artifacts, and communicating clinically relevant findings to the treating team. In this context, pursuing accreditation under the ISO 15189 standard [116] represents a key step toward ensuring consistent quality, reliability, and international alignment in clinical laboratory practice.

Looking ahead, several key trends are anticipated to influence the evolution of MM diagnostics. These trends include the expanded clinical implementation of MRD-guided therapeutic strategies, enhanced automation and digitalization within laboratory workflows, and the advancement of minimally invasive monitoring methods, such as the detection of CTPC. As technologies like NGF and NGS become increasingly accessible and standardized, their adoption into routine clinical practice is expected to rise.

## 5. Conclusions

In conclusion, laboratory methodologies are central to multiple myeloma management, from detection and risk assessment to treatment monitoring and MRD evaluation. Their practical application requires a deep understanding of each method’s strengths, limitations, and appropriate clinical context. With the growth of diagnostic options, laboratories need to have proper technology and expertise to provide results that are accurate, timely, and clinically relevant. Continuous education, quality assurance, and collaboration among clinicians, pathologists, and laboratory scientists are vital to advancing diagnostic excellence and supporting patient care.

## Figures and Tables

**Table 1:** Summary of exams and laboratory parameters that represent the basis of multiple myeloma (MM) diagnosis, classification, and risk stratification.

Sample	Exams/Laboratory Parameters	Purpose/Findings
Peripheral blood (PB)	Complete Blood Count (CBC)	Commonly detects normocytic normochromic anemia; may also reveal leukopenia or thrombocytopenia due to bone marrow infiltration
	Serum β2 microglobulin	Indicates tumor burden and renal function; incorporated into the International Staging System (ISS)
	Serum Calcium	Hypercalcemia is indicative of bone involvement
	Serum Albumin	Low albumin is associated with poor prognosis; incorporated into ISS
	Serum Creatinine	Evaluates renal function, which may be compromised in MM
	Serum Lactate Dehydrogenase (LDH)	High levels may indicate high tumor turnover; incorporated into the 1st Revision of the International Staging System (R-ISS)
	Serum Protein Electrophoresis (SPE)	Screens for the presence of a monoclonal protein (M-protein) appearing as a spike predominantly in gamma or beta region
	Serum Immunofixation Electrophoresis (sIFE)/Immunosubtraction (ISUB)	Identifies the type of monoclonal immunoglobulin (e.g., IgG kappa, IgA lambda)
	Serum Free Light Chain Assay (sFLC)	Quantifies free kappa and lambda light chains; important for diagnosing light chain MM and monitoring disease activity
Urine	Urine Protein Electrophoresis (UPE)	Quantification of MP in a 24 h urine sample
	Urine Immunofixation Electrophoresis (uIFE)	Identifies the type of monoclonal immunoglobulin in urine
Bone marrow (BM)	Cytomorphology (aspirate) and Histology (biopsy)	Confirms diagnosis with ≥10% clonal plasma cells (PC)
	Flow Cytometry	Characterizes the immunophenotype of PC
	Cytogenetics and fluorescence in situ hybridization (FISH)	Detects chromosomal abnormalities with prognostic significance; incorporated into R-ISS and 2nd Revision of the International Staging System (R2-ISS)
	Molecular	Provides deeper genomic profiling of clonal PC

**Table 2:** Most common cytologic features of abnormal plasma cells (PC) and plasmablasts in bone marrow examination. Adapted from [67,68,74,75].

Features	Comments
Abnormal PC	The most common morphology includes: Hyperbasophilic cytoplasm; Rounded eccentric nuclei, whose chromatin looks like a “clock dial” or a “spoked wheel”; Perinuclear colorless zone. Multinucleated PC may be observed. The cytoplasm may contain dense, crystalline inclusions, not exclusive to multiple myeloma, but potentially useful, such as: Clear to colorless inclusions resembling bunches of grapes (morular or Mott cells); Eosinophilic globular inclusions (Russell bodies); “Flaming” red IgA inclusions; Fibrillary Gaucher-like inclusions.
Plasmablasts	Cells with a mismatch between the nucleus and cytoplasm maturation (with a mature cytoplasm but dispersed chromatin and/or a prominent nucleolus). May be morphologically like myeloblasts.

**Table 3:** Summary of the major adverse and favorable prognostic markers in multiple myeloma (MM).

Marker	Gene/Region Affected	Prognosis	Relevance
del(17p13)	*TP53*	Very poor	Found in ~10% of newly diagnosed MM, more frequent in relapsed/refractory disease
t(4;14)(p16;q32)	*FGFR3/NSD2*	Poor	Occurs in ~15% of MM cases; may respond better to proteasome inhibitors
t(14;16)(q32;q23)/ t(14;20)(q32;q11)	*MAF/MAFB*	Poor	Less common (~5% of MM cases); associated with high-risk disease, but prognostic significance debated due to low frequency
+1q21	*CKS1B* and others	Poor	Occurs in ~30–40% of MM cases
del(1p)	Multiple genes	Poor	
del(13q14)	*RB1* and others	Context-dependent	Common (~50%), especially present with t(4;14) (p16;q32); once considered an independent risk factor but now viewed as context-dependent, especially with other high-risk abnormalities
Hypodiploidy	Whole genome	Poor	Associated with aggressive disease
t(11;14)(q13;q32)	*CCND1*	Favorable	Occurs in ~15–20% of MM cases; associated with an indolent disease course and good response to standard therapies (BCL-2 inhibitors)
Hyperdiploidy	+odd-numbered chromosomes	Favorable	Present in ~45–50% of MM cases; often associated with slower disease progression and better response to therapy
Normal cytogenetics	_	_	Absence of high-risk lesions (e.g., del(17p), t(4;14)(p16;q32), +1q21, etc.); indicates standard-risk or low-risk category

## Data Availability

No new data were created or analyzed in this study. Data sharing is not applicable to this article.

## Abbreviations

The following abbreviations are used in this manuscript:

MM	Multiple myeloma
PC	Plasma cells
BM	Bone marrow
MGUS	Monoclonal gammopathy of undetermined significance
SMM	Smoldering multiple myeloma
MP	Monoclonal protein
SPE	Serum protein electrophoresis
sIFE	Serum immunofixation electrophoresis
uIFE	Urine immunofixation electrophoresis
sFLC	Serum free light chains
FC	Flow cytometry
FISH	Fluorescence in situ hybridization
NGF	Next-generation flow
MRD	Measurable residual disease
NGS	Next-generation sequencing
Ig	Immunoglobulins
HC	Heavy chains
LC	Light chains
FLC	Free light chains
rFLC	Free light chain ratio
CE	Capillary electrophoresis
AGE	Agarose gel electrophoresis
PD	Perpendicular drop
TS	Tangential skimming
LOQ	Limit of quantification
IMWG	International Myeloma Working Group
LOD	Limit of detection
MoAbs	Monoclonal Antibodies
ISUB	Immunosubtraction
CR	Complete response
VGPR	Very good partial response
DIRA	Daratumumab-specific immunofixation electrophoresis reflex assay
uIFE	Urine immunofixation eletrophoresis
UPE	Urine protein electrophoresis
dFLC	Difference between involved and uninvolved FLC
HLC	Heavy/light chain
EMN	European Myeloma Network
EHA	European Hematology Association
EDTA	Ethylene diaminetetraacetic acid
PFS	Progression-free survival
OS	Overall survival
CTPC	Circulating tumor plasma cells
CA	Cytogenetic abnormalities
IgH	Immunoglobulin heavy chain
MACS	Magnetic-activated cell sorting
FACS	Fluorescence-activated cell sorting
R2-ISS	Second Revision of the International Staging System
IMS	International Myeloma Society
GEP	Gene expression profiling
SNP	Single-nucleotide polymorphism
DNA	Deoxyribonucleic acid

## Appendix A

**Table A1:** Monoclonal protein detection limits. The table is based on information provided by suppliers and previously published [19,59,117].

Methodology	Analytical Sensitivity ^1^
SPE	1.0 g/L
sIFE	0.1 g/L
sFLC	0.25–3 mg/L
UPE	20 mg/L
uIFE	3–5 mg/L

^1^ The detection limits of monoclonal proteins are influenced by factors previously described, such as their electrophoretic migration pattern and the background level of polyclonal immunoglobulins. Abbreviations: SPE, Serum protein electrophoresis; UPE, Urine protein electrophoresis; sIFE, Serum immunofixation; uIFE, Urine immunofixation electrophoresis; sFLC, Measurement of free light chains in serum.
